# Occurrence and patterns of fin whale songs reveal alternative migration strategies in Svalbard Islands, Norway

**DOI:** 10.1038/s41598-023-31665-x

**Published:** 2023-03-17

**Authors:** E. Papale, M. Pelagatti, G. Pedrazzi, G. Buscaino

**Affiliations:** 1 Institute for the Study of Anthropic Impacts and Sustainability in the Marine Environment (IAS), Unit of Capo Granitola, National Research Council, Via del Mare 3, 91021 Torretta Granitola, TP Italy; 2 Department of Life Sciences and System Biology, University of Torino, Via Accademia Albertina 13, 10123 Turin, Italy; 3 Department of Environmental Biology, Sapienza University of Rome, Piazzale Aldo Moro 5, 00185 Rome, Italy

**Keywords:** Evolution, Zoology, Ecology, Ocean sciences

## Abstract

The Arctic marine environment is highly affected by global warming with notable changes in habitat conditions, which have great consequences on migrating species. For example, the timing of their migration can be altered leading to changes in their occurrence in suitable areas, which are critical for their survival. In this study, seven years of acoustic data were analysed in Svalbard Islands from 2014 to 2020, revealing that the occurrence of fin whales (*Balaenoptera physalus*) happened all year-round. The sea surface temperature recorded reveals conditions which could be favorable for these species to persist until the Polar Night. The occurrence of songs indicated that certain individuals did not undertake the migratory journey through the southern breeding grounds, possibly using the area for mating purposes. The analyses of the Inter-Note-Interval (INI) demonstrated that over the years songs with different patterns were found. This suggests that either the fin whales are able to switch their INI patterns or that populations with different INIs are visiting during the Winter. Therefore, this study unveils the undertaking of an alternative strategy to migration movements, and the possible potential origin of the fin whales overwintering in Svalbard.

## Introduction

The Arctic is drastically changing due to global warming and anthropogenic activities^1^. Current alterations are influencing the temporal and spatial habitat suitability, forcing species to rapidly adapt to modifications of the environment and the food web^2,3^. Marine mammals are sentinels of these changes because their presence, behaviour, and fitness are tightly linked to ecosystem conditions^4^.

Fin whales (*Balaenoptera physalus*) are known to migrate to high-latitude regions to feed on krill and small-sized pelagic fish during summer months and move southwards to temperate regions to breed during winter^5^. They are mostly absent in areas where sea ice concentration is high, showing a negative correlation between their presence and ice coverage^6–8^. Furthermore, recent modeling studies revealed that fin whales prefer a narrow range of sea surface temperature (between 4 and 7 °C, peaking at around 6 °C)^9,10^ and low seasonal temperature variability^7^.

Along the west coast of Svalbard, sightings of this species have been recorded up to 81.5° N from March to November, with peaks in June–September^5^. The Western Svalbard coastal waters are characterized by the influence of the warm and salty North Atlantic Current and colder Arctic water masses^11^. Starting in the early 2000s, the strong influence of the northernmost part of the North Atlantic Current, which enters the West Spitsbergen Current, kept waters void of sea ice^12^ and carried boreal species to northern latitudes^2^. From 2002 to 2019, fin whales’ sighting rates increased in the coastal environment and inside the fjords of the archipelago^13^. They showed a poleward shift after 2010, particularly in northern latitudes concomitant with the increase of intrusion of warmer waters and the newfound abundance of their Atlantic prey species^5,14,15^. Through satellite tagging, Lydersen et al.^9^ noted that in the period 2015–2019 some fin whales persisted in the area, at least until the onset of the Polar Night, feeding around Svalbard and in the Fram Strait. Intermittent and low-intensity fin whale calls were also recorded during winter in the Fram Strait from 2009 to 2010^16,17^, thus suggesting changes in fin whale distribution in the last years.

Due to the changing environmental conditions and the cues showing that fin whales persist at high latitudes along the Polar Night, the knowledge of when and how whales frequent Arctic areas is crucial for their conservation in the current framework of climate change.

Passive Acoustic Monitoring (PAM) is a cost-effective monitoring tool for detecting whale distributions over large areas and multiple years, especially when visual surveys are unfeasible. It is particularly valuable to recognize the timing of migration and temporal trends, which is critical for improving management and mitigation efforts^18^. Fin whales mainly produce songs formed by downsweep stereotyped notes ranging from 25 to 15 Hz, in about 0.5–1 s, usually centered at 20 Hz, and are distributed worldwide[^6,19–28^ among the others]. The 20 Hz notes can also be irregularly produced when animals are in group, probably serving a social function^29–31^. All biopsied singing whales have been identified as males^23^, and the overlap with the reproductive period suggests that they may use songs as a male acoustic display. Other vocalizations emitted during songs are the backbeat, low-frequency signals remaining relatively constant in duration (about 0.8 s) and frequency (18–20 Hz)^22^, and a higher upsweep frequency note at about 130 Hz^6,32^. Backbeats usually alternate with 20 Hz notes^20,27^, while the higher frequency component can be produced especially during the reproductive period^31^.

The 20 Hz note sequences usually present regular inter-note intervals (INIs). These sequences can be separated by short (1–20 min) or long gaps (20 min–2 h)^27,33,34^. INI patterns may remain constant (singlet patterns), or vary within a song (doublet or triplet)^19–21,35,36^. Furthermore, INI may change over seasons^37,38^, or shift over long periods of time^36,39^. Several studies indicate that stable regular INI patterns may be used for stock differentiation^22,33,36,40^, and can reveal migration and distribution patterns in this species^40^.

Here, acoustic data were used to infer acoustic presence and song patterns of fin whales in the area. In particular, to investigate (1) the temporal occurrence of fin whale calls; (2) the relationship between call rates and sea surface temperature (as a cue of environmental conditions)^41^; (3) the song structure and INI patterns as proxies of the presence of differing acoustic populations or song changes.

## Materials and methods

### Survey area and data collection

**Figure 1:**
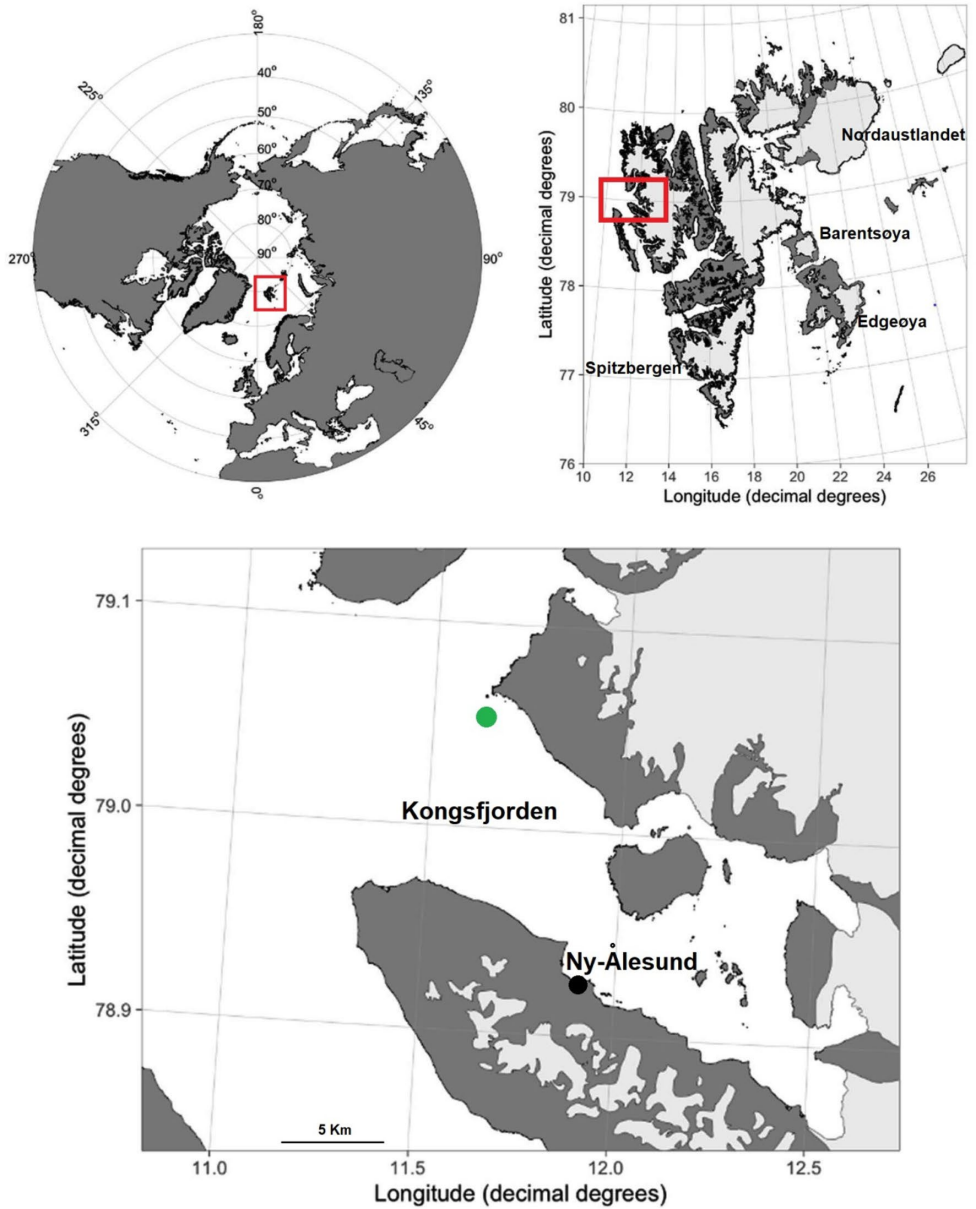
Location of Kongsfjorden in Svalbard Islands and of the acoustic recorder (green dot). The map was generated in R package version 0.9.2^62^.

Data were collected in the Kongsfjorden, on the western side of Spitsbergen Island, in the Svalbard archipelago (79° N–12° E, Norway) (Fig. 1). An underwater autonomous passive acoustic recorder (SM2, Wildlife Acoustics, US) was deployed from 2014 to 2020 at the mouth of the fjord (79°03.22′ N–11°32.83′ E) at about 75 m from the surface, in an area with maximum depths of 83 m^42–44^. The omnidirectional calibrated hydrophone had a sensitivity of − 165 ± 5 dB re 1 V/μPa from 2 Hz to 30 kHz. The sampling frequency was set at 48 kHz, with a 16 bit resolution A/D converter. The duty cycle was set at 50%: recording the first 30 min of every hour. Due to a failure of the system, only a couple of minutes per hour were collected during 45 days in 2019, and 15 days in 2020 (Supplementary Materials—SM1).

### Data analysis

#### Temporal patterns

Recordings were downsampled to 500 Hz and amplified 14 dB by using MatLab software (R2021a, Mathworks). An energy-band based automatic detector, that measures the amount of energy in a specific frequency band of the spectrogram, was used in Ishmael 3.0^45^ to select fin whale songs. The detector was set by selecting the signal frequency range of interest (18–25 Hz), the minimum and maximum duration (0.1–2.5 s) and the minimum time between consecutive signals (Neighbourhood) (2.5 s). The “energy ratio” and the “equalizations” tools were used to limit the range of frequencies examined and reduce long background noise. In this case, 25–100 Hz and 0.5 s was used. Furthermore, the “smoothing” tool smoothed the differences of energy sum. Spectrogram parameters were adjusted to improve signal display with the following settings: Frame size = 1024, zeropadding = 1×, hop size = 1/8×. Based on a manual comparison of a 3 months data subset, the software setting selected provided a high value of accuracy (95%) and sensitivity (75%) (Supplementary Materials−SM2). Since false negative rate was 0.52%, and false positive rate 4.42% due to noise from ice and anthropogenic activities, all recordings with detections were manually checked to avoid any false positives. Data were amplified again (in 9.5 dB) to increase whale signals’ detectability and spectrograms visualized using RX 3.0.2.812 (iZotope Inc., 2013) (Hamming window, FFT = 1024, overlap = 87.50%).

To investigate acoustic presence, the Presence Rate (PR) was calculated on the 20 Hz notes (and on the 130 Hz notes when they were emitted alone) as:

$$\frac{\mathrm{number \,of \,recordings\, with \,fin \,whale \,detections \,per \,week}}{\mathrm{number \,of \,recordings\, per\, week}}$$

while to examine the rate of vocalizations, the Detection Rate (DR) on the 20 Hz notes (and on the 130 Hz notes when they were emitted alone) was calculated as:

$$\frac{\mathrm{number\, of\, calls\, per\, week}}{\mathrm{recorded \,minutes\, per \,week}}$$

Presence Rate was used as a proxy of presence of vocalizing animals, and Detection Rate gives clues on vocal behaviour, the intensity of singing.

Generalized additive models (GAM) (R “mgcv” package version 1.8–28;^46^) have been used to model both seasonal patterns and long-term trends. PR and DR were tested as a function of the predictors *Month* and *Year*. A gaussian distribution and an identity link function were chosen. Cyclic cubic and cubic regression splines were respectively used for the explanatory variables. All statistical analyses were performed in R 2022.02.0^47^.

#### Sea surface temperature

Daily sea surface temperature from satellite data were downloaded from Copernicus Marine Service (https://marine.copernicus.eu/), from January 2014 to February 2020. They were processed by using Anaconda Prompt (Anaconda 2022.10, ©Anaconda Inc.). Mean temperatures and standard deviations were calculated for each week. To estimate a relationship between sea surface temperature and PR and DR, non-parametric Spearman’s correlation tests for not normally distributed data were performed. Correlation was considered weak at rho value < 0.39, while strong at rho value > 0.60.

#### Song structure analysis

**Figure 2:**
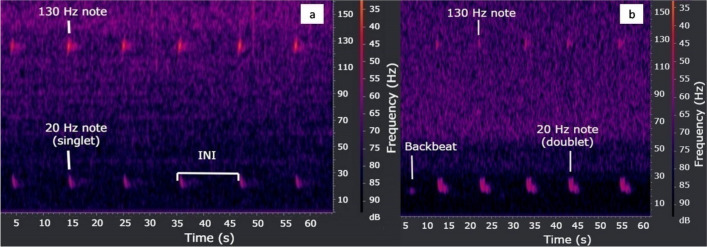
Spectrograms (Hamming window, FFT = 1024, overlap = 87.5%) of fin whale songs consisting of (**a**) 20 and 130 Hz notes indicating the inter-Note-Interval (INI), measured from the beginning of a note to the beginning of the following note of the same type, and (**b**) 130 Hz notes, doublet 20 Hz notes and backbeats. Both spectrograms were generated with RX 3.0.2.812.

Songs were considered as series of sequences of 20 Hz notes with a regular interval, separated by periods of silence, hereafter Rests. INIs were manually measured in the spectrogram as the time interval between the beginning of a note to the beginning of the following one, as shown in Fig. 2^20^. Inter-note intervals selection for the song structure analysis was performed examining all songs recorded from September to April (considered the singing season). Only a couple of 20 Hz note sequences with a regular interval were collected during August, but due to the low quality of the recordings, they were not analysed.

Since the observed 130 Hz note INIs matched the one of the 20 Hz notes, the INIs used for analysis were measured between 20 Hz notes, and between 130 Hz notes when songs were made up of only the high frequency component.

Then, only songs containing more than 10 notes were selected for analysis. At least 7 INIs, up to 20 INIs per song, were selected (Table1).

**Table 1:** Number of songs and INIs analysed per month and singing season.

Season	Month	# Songs 20 Hz	# Songs 130 Hz	# INI *per* Song (Min–Max)
2014/15	Sept	1	0	18
	Oct	5	1	19–20
	Nov	1	0	20
	Jan	0	1	20
2015/16	Oct	0	1	20
	Nov	1	0	7
	Jan	1	0	20
2016/17	Apr	2	0	20
2018/19	Oct	1	2	20
	Nov	1	2	20
2019/20	Oct	0	2	20
	Nov	1	3	9–20
	Dec	1	0	7

To limit the risk of non-independence of data, INIs belonging to different songs were selected in recordings at least 24 h apart from each other. INIs corresponding to or occurring during Rests were not included.

## Results

### Overall

In total, 4774 h of recordings were analysed. The acoustic presence of fin whales was detected in 387 h, and 16,014 signals were counted. Three kinds of notes were observed: 20 Hz notes, backbeats and 130 Hz notes (Fig. 2). Backbeats and 130 Hz notes were found only within songs. However, due to the low number of backbeats, only 20 and 130 Hz notes were considered for analyses. The 130 Hz notes have often been found together with the low frequency component, except for 67 recordings, where they have been found alone. In these recordings, they were probably produced alone because low levels of noise were recorded at frequencies up to 50 Hz^32^.

**Figure 3:**
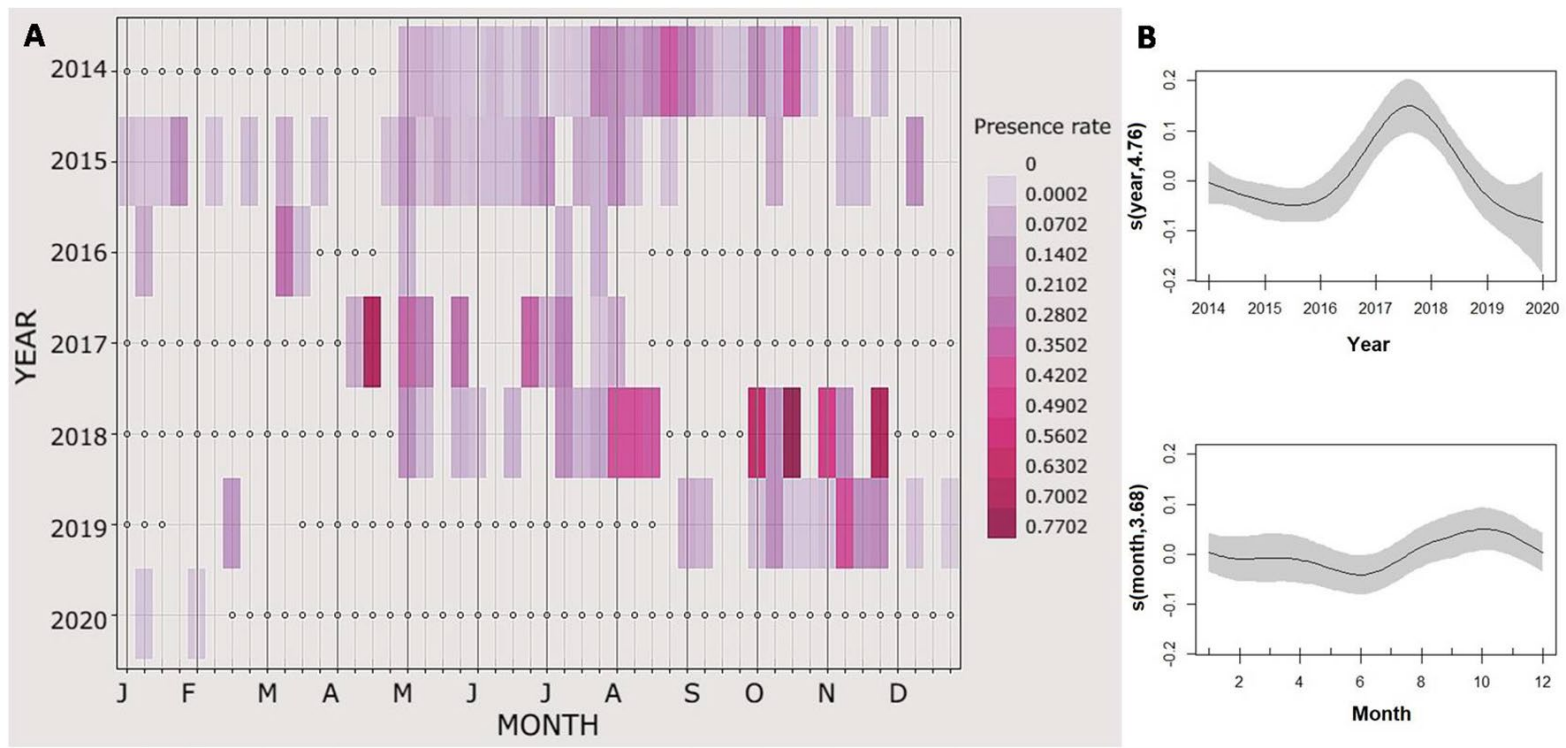
(**A**) Fin whale acoustic presence per week (presence rate) along the 7 years considered. White dots represent absence of data. (**B**) Response curves of the variables in the Generalized Additive Models (GAMs) performed testing PR as a function of the predictors Year and Month. The solid lines represent the smoothed estimates by the GAM, while the grey areas represent the approximate 95% confidence intervals.

Fin whales were detected all years and throughout the year, even if the gaps in the recordings should be considered (Fig. 3). GAM analysis showed that the predictor variables *Year* and *Month* explained the 25.6% of the PR model deviance, with the variable *Year* as the major contributing factor (p < 0.001) over the variable *Month* (p < 0.01). Indeed, an inconsistent seasonal pattern of vocal presence was identified over the years of recording. PR showed the highest peak in 2018 compared to the other years (Fig. 3, Supplementary Materials−SM3).

**Figure 4:**
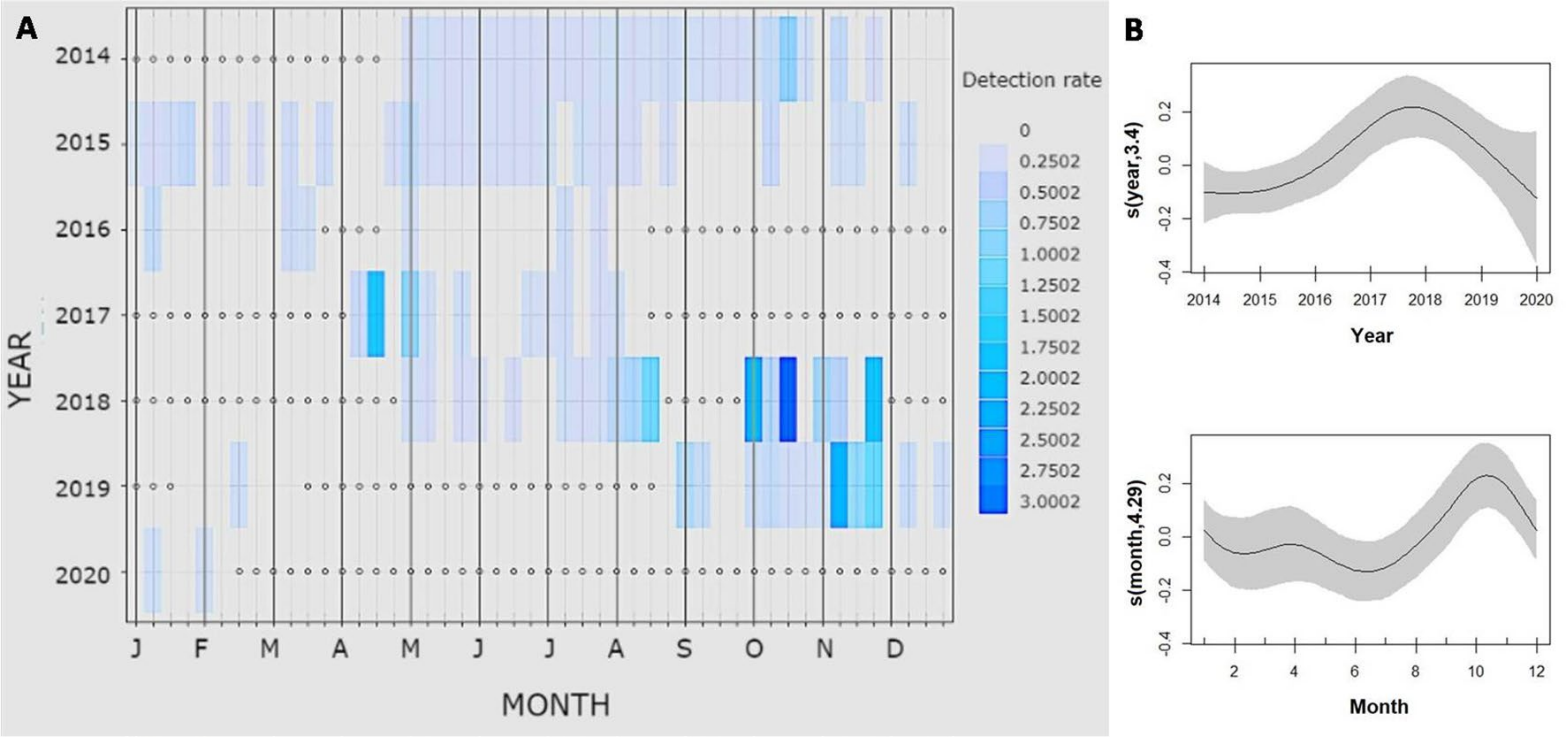
(**A**) Fin whale calls per week (detection rate) along the 7 years considered. White dots represent absence of data. (**B**) Response curves of the variables in the Generalized Additive Models (GAMs) performed testing DR as a function of the predictors Year and Month. The solid lines represent the smoothed estimates by the GAM, while the grey areas represent the approximate 95% confidence intervals.

*Year* and *Month* both contributed as predictor variables to DR model variation (p < 0.001, 22.8% deviance explained). DR was higher during October and November (Fig. 4, Supplementary Materials—SM3). Comparing DR at the beginning of the Polar Night, in October, with the rest of the year, an increase in the calling rate was recorded (Mann Whitney test Z = − 3.02, p = 0.003), while PR did not show any differences.

### Sea surface temperature

**Figure 5:**
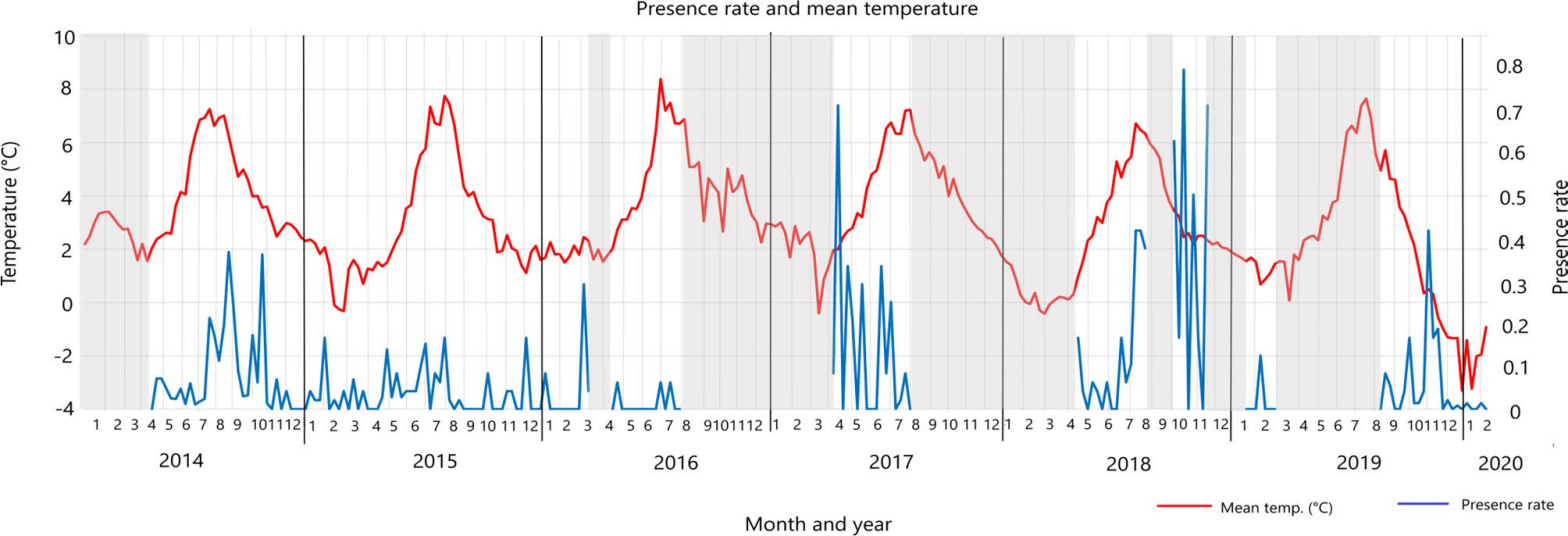
Weekly fin whale presence rate (in blue) and mean sea water temperature (in red). Light grey strips represent periods with no data.

Figure 5 shows the sea surface temperature averaged by weeks from January 2014 to February 2020, and the weekly PR. Sea surface temperatures fluctuated between 1 and 4 °C from October to May, except for a couple of weeks in February 2015, one in March 2017, and four in February–March 2018, when mean sea surface temperature reached 0 °C. Only in December 2019 and the first months of 2020, sea surface was covered by ice. From the beginning of June to the end of September, sea surface temperature fluctuated between 4 and 8 °C.

A positive, but weak, correlation was found between PR and sea surface temperature (Spearman S = 730,064, p < 0.05, rho = 0.21), while DR was not related (S = 867,323, p = 0.42). The highest PR values were reached at a sea surface temperature range of 2–7 °C, peaking at 6–7° in July–August, and at 2–3° in Spring and Fall.

### Song structure

**Figure 6:**
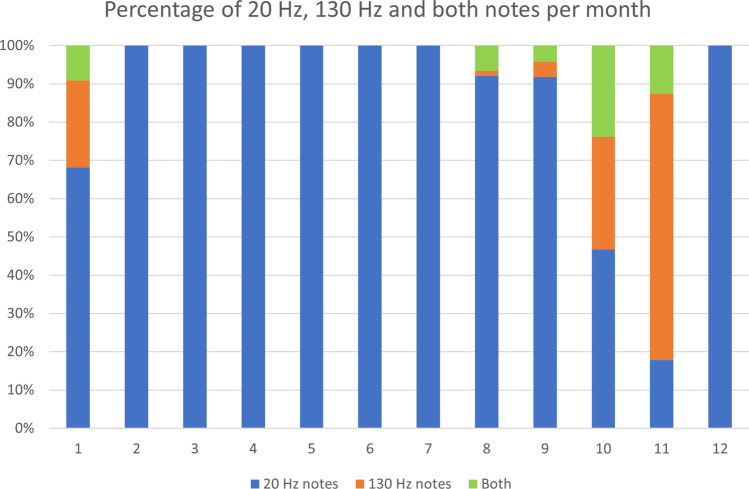
Histogram representing relative percentages of 20-Hz and 130 Hz per month (1–12) over the entire dataset.

The 30% of the songs were made up of 20 Hz and 130 Hz notes simultaneously. 130 Hz notes were present every year, mainly in October and November followed by September and January, except for 2017 when data were collected only in Spring and Summer months (Fig. 6).

**Figure 7:**
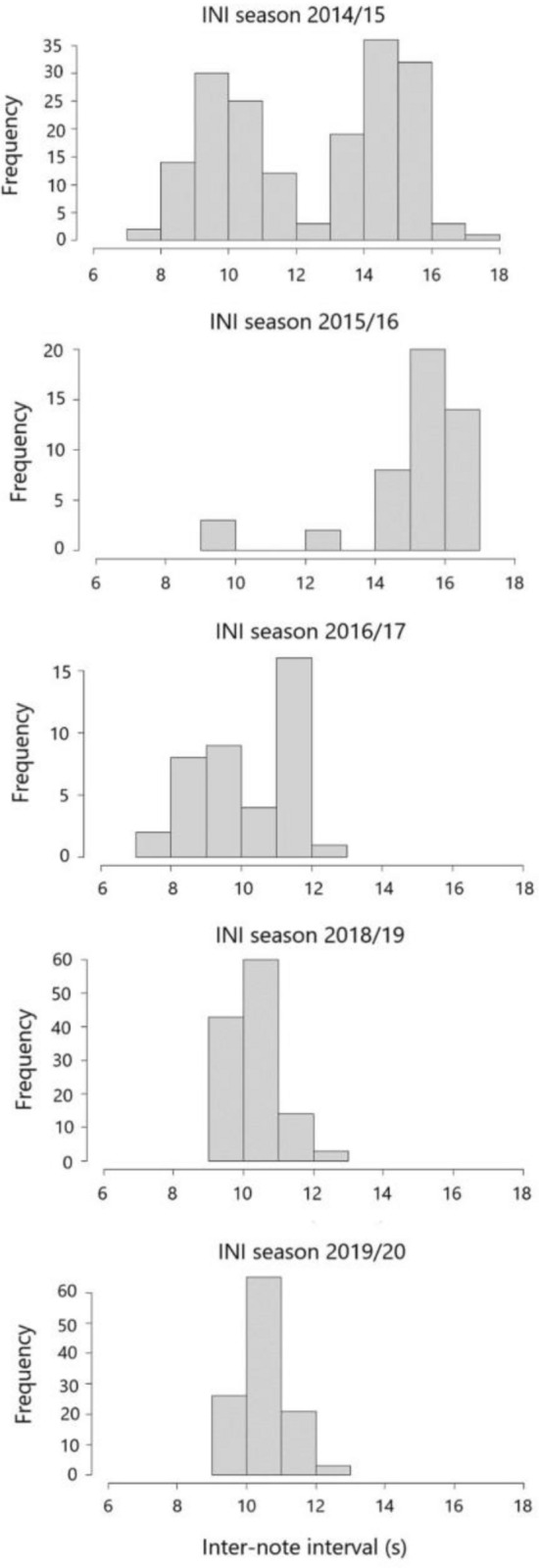
Inter-note-interval distribution along the singing seasons (from September to April) recorded.

The analysis of the INIs performed on both kind of notes (20 Hz and 130 Hz) revealed that, in the singing season of 2014–2015, two kinds of patters occurred: one bimodal with INIs at 9 and 14 s, and one containing only ~ 15 s INIs. The percentage of bimodal songs (including long and short INIs) was 67%. In the following season (2015–2016), only unimodal 15 s songs were detected, while in the next years INIs were shorter (~ 10 s songs). Indeed, in 2016–2017, two songs were recorded, at the beginning of April, with the first song having longer INIs compared to the second (mean 11.30 ± 0.43; mean 9.04 ± 0.90). In 2018–2019 and 2019–2020, however, INIs slightly increased, and remained almost unvaried between 9 and 12 s (Table 2, Figs. 7, Supplementary Materials—SM4).

**Table 2:** Notes and INI patters along the singing seasons considered.

Singing season	Notes	Bimodal INI (mean ± SD)	Unimodal INI (mean ± SD)
2014–2015	20 Hz	11.92 ± 2.40 s	14.66 ± 0.99 s
	130 Hz	10.51 ± 2.35 s	15.42 ± 0.29 s
2015–2016	20 Hz		15.08 ± 2.30 s
	130 Hz		15.13 ± 0.42 s
2016–2017	20 Hz		10.17 ± 1.34 s
2018–2019	20 Hz		10.31 ± 0.54 s
	130 Hz		10.35 ± 0.65 s
2019–2020	20 Hz		10.81 ± 0.41 s
	130 Hz		10.57 ± 0.63 s

Both 20 Hz and 130 Hz notes INIs were significantly different among singing seasons (respectively Kruskal–Wallis χ^2^ = 67.01, p-value < 0.001; χ^2^ = 61.58, p-value < 0.001). In particular, they were both higher in the 2015/16 season compared to the other singing seasons (Supplementary Materials−SM5).

Rests occurred in every song, and were shorter (mean = 17.73 s, SD = 1.00) when INIs were short (around 9 s), but increased in duration (mean = 32.50 s, SD = 2.63) with longer INIs (15–16 s).

Finally, backbeats were found only in two songs recorded during 2018–2019 and 2019–2020. The first, in October 2018, contained 47 backbeats and the other in September 2019 had only 3 backbeats. In the same song of the 47 backbeats, also 20 Hz note *doublet* occurred (Fig. 2b).

## Discussion

Fin whales were acoustically present throughout the entire study period with a relative year-to-year variability.

Regarding the acoustic presence (PR), no seasonality was found in the models. Although this could be the result of data gaps, data from a complete year in 2015 does not reveal any seasonality. The summer acoustic presence of fin whales is consistent with Storrie et al.^5^ findings. Their absence in September 2015, may be linked to variability in their migration timing, since, in the other years, calls were always detected during that month. Lydersen et al.^9^ found that some fin whales remain at high latitudes until the onset of the Polar Night. Our data concurs with this outcome as the species was acoustically present in October and November for all the years recorded. Furthermore, results indicated that they remained in the area during Winter.

Baleen whales have already been detected to display shifts in migration timing or alterations due to climate change^18,48^. Rising water temperatures can lead to changes in prey species’ presence and availability. Since whale movements at high latitudes can be driven by prey hotspot seasonality^49,50^, the recent northward range expansion^5^ and the permanence during Winter can be supported by the increase of krill^51^ and boreal fish species at higher latitudes^44^. Moreover, fin whales generally prefer areas with low sea ice concentrations and homogenous sea surface temperatures lower than 10 °C^7^. Data demonstrated that these temperatures have been recorded also during the period when the species typically begins its migration in September/October. Specifically, temperatures remained above the 4 °C threshold until October. Until at least January, temperatures dropped between 4 and 2 °C without abrupt variations^7^. The sea surface was covered by ice only in the winter season of 2019–2020, where sea temperatures were recorded at lower than − 2 °C. The correlation between sea surface temperature and PR, although weak and only marginally significant, validates the hypothesis of a preference for this temperature range. The reduction in sea ice coverage and the increase in sea surface temperature caused by climate change could improve habitat suitability, forcing fin whales to postpone their departure, or shift the temporal permanence in the area.

The DR instead showed no relations with sea surface temperature, highlighting that the increase in calls is not due to temperature, but rather to a shift in the acoustic behavior. Results suggest that at the onset of the Polar Night (in October), a significant increase in call detection rate occurs in Svalbard Islands. The increase can be explained by the change in fin whales’ calling behavior which switches from irregular 20 Hz notes to song-forming 20 Hz notes. Songs have been consistently recorded in the area until the end of January, drastically decreasing after, with the last songs recorded at the beginning of April (in 2017). As songs are only carried out by mature males^23^, there is a limitation in the portions of the population being detected because it is not possible to establish if immature males^52^, and females are non-migrating individuals.

Songs analysed here consisted of mainly 20 and 130 Hz notes, but both notes were also found on their own. As suggested by Garcia et al.^32^, fin whales can voluntarily produce these vocalization types, either individually or in combination, as a strategy linked to the propagation conditions. However, the potential influence that the distance to the source has on the detection of either note was not evaluated in this work, and therefore, it is not possible to confirm this hypothesis here.

The analysis of INI patterns collected in Svalbard waters from 2014 to 2020 (Fig. 7) showed that changes occurred over the years. Song of fin whales can provide information about its geographic areas of origin^22,53^. Among all song properties, INIs have been considered as a cue to stock differentiation^22,33,36,53^. However, the same population of fin whales can sing many different song patterns, both within a season and between seasons^28,37–39,54^. Therefore, two hypotheses can be put forward: the first is that fin whales belonging to the same acoustic stock switch INIs, the second states that there is a year-by-year change in whales’ acoustic stocks.

The first hypothesis suggests that fin whales can sing different patterns and new songs can emerge^36^. In this case, a cultural transmission between specimens that share the same feeding areas has been proposed as explanation^28,39,54^.

The second hypothesis instead suggests that non-migrating fin whales may come from different acoustic stocks. Furthermore, it also implies, in this situation, that whales persisting at higher latitudes during one breeding season might choose to migrate southward the following year and vice-versa. Such adaptive change of behavior suggests that fin whales possess the plasticity to change their migration strategies. However, neither of the two hypothesis can be confirmed because of the lack of concurrent fin whales song INI data from other regions.

The INI patterns recorded showed bimodal songs made up of a single note type, in 2014–2015. A similar pattern was recorded in the Gulf of Maine in 2006 and 2007^33^. The means of the two INI types ranged at 8–10 s and 14–16 s, corresponding to the bimodal pattern recorded after several years in Svalbard Islands (Fig. 7). However, given that INIs show gradual variations over the years^36,39,55^, a clear connection cannot be established.

In the same and the subsequent singing season, unimodal songs with a 15–16 s INI pattern were recorded. A similar song structure could be emitted by conspecifics coming from southeastern areas of the Atlantic, such as the Bay of Biscay and the South of Portugal^27^, where a pattern made up of INIs ranging from 14 to 16 s was recorded during the singing seasons in 2007–2008 and in 2008–2009. Pereira et al.^27^ suggest that it might belong to individuals coming from the Mediterranean, since Castellote et al.^56^ recorded a similar INI pattern. This was recently confirmed by Morello^57^, that found INIs averages ranging 14–17 s in 2012–2013 in the Central Mediterranean, and by Best et al.^58^, that found a range from 15 to 16.5 s in 2014–2017 in the Northwestern Mediterranean. The migration route to the Southern Portugal and Moroccan waters has already been detected by Lydersen et al.^9^ in November 2015 and 2019 through satellite tracks. The individuals recorded passed along Norway, Ireland, and coastal Portugal. Another confirmation for this route was a song with INIs at 15.3 s, attributable to an individual moving in southern Norway in February 2014^32^.

In the season 2016–2017, songs with shorter INIs compared to those of the previous years were recorded in Svalbard. The range was between 7.5 and 12 s. These values are too low to be attributable to fin whales in the Eastern North Atlantic in the same period^59^.

However, in 2018–2019 and 2019–2020, unimodal INIs slightly increased compared to the previous year, and remained almost unvaried between 9 and 12 s. Asimilar pattern could be found in the Western North Atlantic: in particular, in Massachusetts Bay and New York Bight regions the average short INI pattern was 9.6 s^37^, from 2008 to 2010; in the Gulf of Maine where INI length was around 9 s in 2006–2007 and 2007–2008; and in the Gulf of Saint Lawrence, where it was 11–12 s in 2005–2006^35^. However, the shift in the INI patterns for the Massachusetts Bay and New York Bight regions was calculated as 0.5 s/year^37^, so it is not possible to attribute with certainty the origin of the whales calling in 2018–2020.

The presence of backbeats in 2018–2020 suggests the visiting of Svalbard by individuals from other acoustic stocks. Indeed, in 2014 Garcia et al.^32^ detected in Norwegian and Barents Sea waters songs with both backbeats, and 20 Hz notes singlet and doublet. Since in our dataset only two examples of a similar song were found, it is likely to be two northward visitors. However, the INI between the 20 Hz notes forming the doublet changes along the song from 3.24 to 0.80 s, suggesting it could represent a multipath^24,29,39,60^.

These outcomes leave the door open to both the explanations highlighted before: the presence of whales from different areas, and the possible cultural emergence of new songs or shifts in patterns. Therefore, further studies are needed to understand the changes over time and to provide information on the species presence in a changing Arctic.

## Conclusions

Outcomes from this work suggest that not all fin whales migrate, and an increase in acoustic presence may be caused by more whales migrating North during the summer, as corroborated by satellite tag data.

Furthermore, differences in INIs may reflect either fin whales switching INIs or different acoustic populations visiting the area. If the second hypothesis were true, it would indicate that individuals change their migration patterns over the years. Fin whales may adopt different strategies of resource tracking to foresee the best areas for feeding^61^. Therefore, local studies in hotspot areas could be useful for the species at a global level, considering that the fin whale range shows almost a world-wide extension. Constant monitoring over a long period could indeed help in identifying how these species are responding to environmental and anthropogenic changes and could provide support in developing management plans for crucial species in a changing Arctic.

## Supplementary Information

Supplementary Information.

## Data Availability

Correspondence and requests for materials should be addressed to E.P.
