# Supplementary material for: Occurrence and patterns of fin whale songs reveal alternative migration strategies in Svalbard Islands, Norway
Source: Sci Rep. 2023 Mar 17;13:4436. doi: 10.1038/s41598-023-31665-x (PMC10023778; doi:10.1038/s41598-023-31665-x)
Supplement: Supplementary file 1 — Supplementary Information. [file 41598_2023_31665_MOESM1_ESM.pdf]

Occurrence and patterns of fin whale songs reveal alternative migration strategies in Svalbard Islands, Norway

Papale E.[ab*], Pelagatti M.[a], Pedrazzi G.[c], Buscaino G.[a]

[a] *Institute for the Study of Anthropic Impacts and Sustainability in the Marine Environment (IAS), unit of Capo Granitola, National Research Council, Via del Mare 3, 91021 Torretta Granitola (TP), Italy*

[b] *Department of Life Sciences and System Biology, University of Torino, Via Accademia Albertina 13, 10123 Torino, Italy*

[c] *Department of Environmental Biology, Sapienza University of Rome, Piazzale Aldo Moro 5, 00185 Rome, Italy*

SM1 Detail of the recording schedule and hours recorded per each year from 2014 to 2020

<table>
<tr><th></th><th colspan="2">2014</th><th colspan="2">2015</th><th colspan="2">2016</th><th colspan="2">2017</th><th colspan="2">2018</th><th colspan="2">2019</th><th colspan="2">2020</th></tr>
<tr><th></th><th>Recording schedule</th><th>Hours recorded (h, m)</th><th>Recording schedule</th><th>Hours recorded (h, m)</th><th>Recording schedule</th><th>Hours recorded (h, m)</th><th>Recording schedule</th><th>Hours recorded (h, m)</th><th>Recording schedule</th><th>Hours recorded (h, m)</th><th>Recording schedule</th><th>Hours recorded (h, m)</th><th>Recording schedule</th><th>Hours recorded (h, m)</th></tr>
<tr><td>January</td><td colspan="2" rowspan="3">no data</td><td>30/60 (50%)</td><td>131.5</td><td>30/60 (50%)</td><td>60</td><td colspan="2" rowspan="3">no data</td><td colspan="2" rowspan="4">no data</td><td>30/60 (50%)</td><td>9.5</td><td>2/60 (3.3%) 30/60 (50%)</td><td>28.8</td></tr>
<tr><td>February</td><td>30/60 (50%)</td><td>108</td><td>30/60 (50%)</td><td>60</td><td>30/60 (50%)</td><td>48</td><td>2/60 (3.3%)</td><td>2.6</td></tr>
<tr><td>March</td><td>30/60 (50%)</td><td>120.5</td><td>30/60 (50%)</td><td>36</td><td>30/60 (50%)</td><td>24</td><td colspan="2" rowspan="10">no data</td></tr>
<tr><td>April</td><td>30/60 (50%)</td><td>14.5</td><td>30/60 (50%)</td><td>120</td><td>30/60 (50%)</td><td>36</td><td>30/60 (50%)</td><td>36</td><td colspan="2" rowspan="4">no data</td></tr>
<tr><td>May</td><td>30/60 (50%)</td><td>372</td><td>30/60 (50%)</td><td>113.5</td><td>30/60 (50%)</td><td>108</td><td>30/60 (50%)</td><td>48</td><td>30/60 (50%)</td><td>84</td></tr>
<tr><td>June</td><td>30/60 (50%)</td><td>372</td><td>30/60 (50%)</td><td>120</td><td>30/60 (50%)</td><td>96</td><td>30/60 (50%)</td><td>48</td><td>30/60 (50%)</td><td>72</td></tr>
<tr><td>July</td><td>30/60 (50%)</td><td>334</td><td>30/60 (50%)</td><td>132</td><td>30/60 (50%)</td><td>120</td><td>30/60 (50%)</td><td>48</td><td>30/60 (50%)</td><td>72</td></tr>
<tr><td>August</td><td>30/60 (50%)</td><td>372</td><td>30/60 (50%)</td><td>108</td><td>30/60 (50%)</td><td>48</td><td>30/60 (50%)</td><td>24</td><td>30/60 (50%)</td><td>60</td><td>2/60 (3.3%)</td><td>2.4</td></tr>
<tr><td>September</td><td>30/60 (50%)</td><td>360</td><td>30/60 (50%)</td><td>36</td><td colspan="2" rowspan="4">no data</td><td colspan="2" rowspan="4">no data</td><td colspan="2">no data</td><td>2/60 (3.3%) 30/60 (50%)</td><td>21.6</td></tr>
<tr><td>October</td><td>30/60 (50%)</td><td>180</td><td>30/60 (50%)</td><td>60</td><td>30/60 (50%)</td><td>48</td><td>2/60 (3.3%) 30/60 (50%)</td><td>21.6</td></tr>
<tr><td>November</td><td>30/60 (50%)</td><td>120</td><td>30/60 (50%)</td><td>60</td><td>30/60 (50%)</td><td>48</td><td>2/60 (3.3%) 30/60 (50%)</td><td>40.8</td></tr>
<tr><td>December</td><td>30/60 (50%)</td><td>120.5</td><td>30/60 (50%)</td><td>72</td><td colspan="2">no data</td><td>2/60 (3.3%) 30/60 (50%)</td><td>20.8</td></tr>
</table>

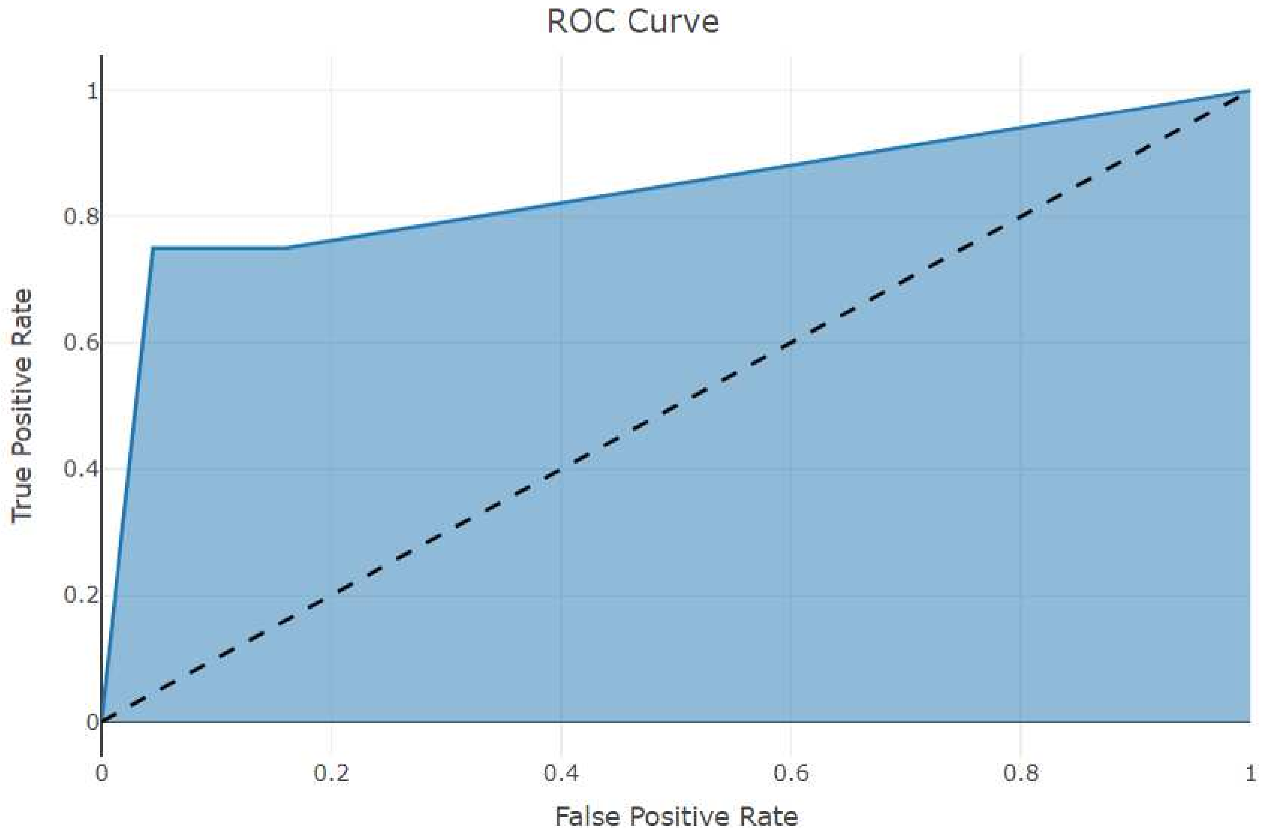

SM2 Roc curve (plot of the true positive rate (Sensitivity) in function of the false positive rate) showing the performance of the different tested settings of the software.

	Generalized Additive Models			
	**Explanatory variables**	**F**	**p-Value**	**Deviance explained**
**PR**	**Month**	1.03	0.01	25.6%
	**Year**	7.95	<0.001	
**DR**	**Month**	2.01	<0.001	22.8%
	**Year**	4.37	<0.001	

SM3 Results of Generalized Additive Models (GAMs) performed testing PR and DR as a function of the predictors Month and Year.

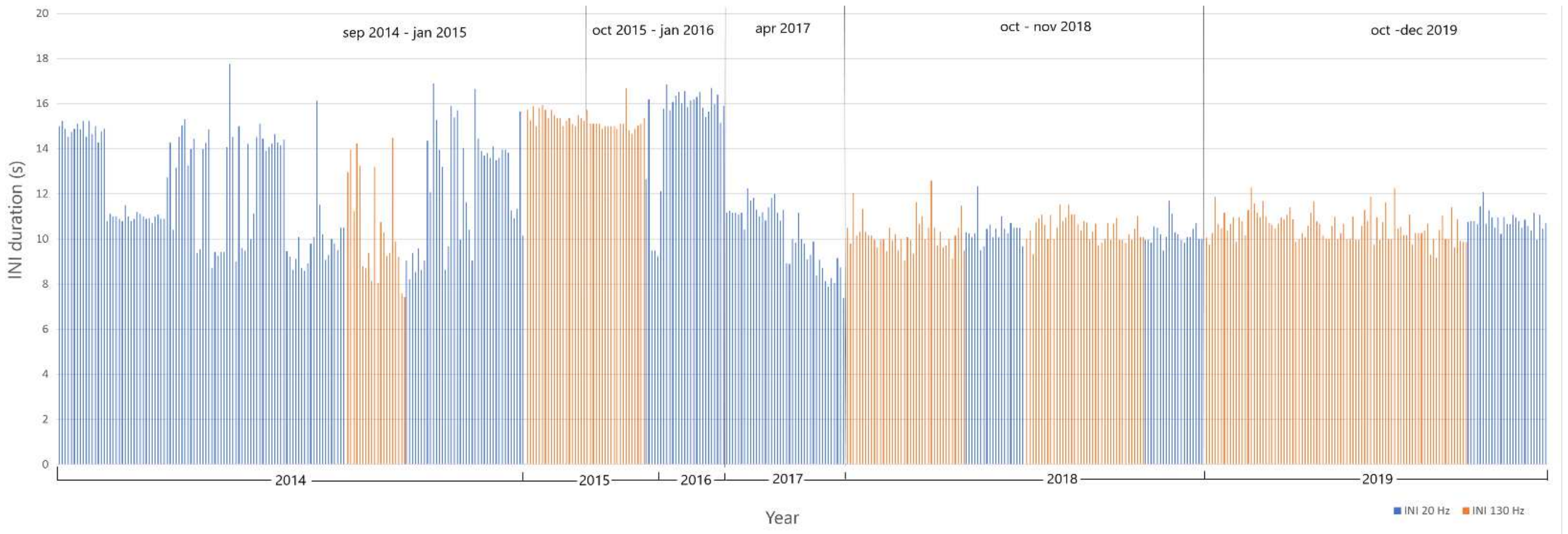

SM4 Distribution of INI duration along the songs per year and singing seasons (INI calculated on 20Hz notes in blue, INI measured on 130 Hz notes in red)

a)

20 Hz note	2014/15	2015/16	2016/17	2018/2019
2015/16	**<0.001**	-	-	-
2016/17	**<0.001**	**<0.001**	-	-
2018/19	**<0.001**	**<0.001**	0.99	-
2019/20	**<0.001**	**<0.001**	0.06	**<0.001**

b)

130 Hz note	2014/15	2015/16	2018/2019
2015/16	**<0.001**	**-**	-
2018/19	**<0.001**	**<0.001**	-
2019/20	**<0.001**	**<0.001**	0.17

SM5 Differences between INIs recorded along the singing seasons: results of theTamhane post hoc test performed on a) 20 Hz INI; b) 130 Hz INI. Significant p-values are in bold.
